# The Potential of Cytokinins as Innovative Components of Multifunctional Biomaterials: Mechanisms of Biological Activity and Potential in Tissue Regeneration—In Vitro Studies—Review

**DOI:** 10.3390/cimb48101010

**Published:** 2026-10-01

**Authors:** Agata Jabłońska-Trypuć

**Affiliations:** Department of Chemistry, Biology and Biotechnology, Faculty of Civil Engineering and Environmental Sciences, Bialystok University of Technology, Wiejska 45E Street, 15-351 Bialystok, Poland; a.jablonska@pb.edu.pl

**Keywords:** cytokinins, kinetin, benzyladenine, traumatic acid, biomaterials, regeneration, in vitro, skin, human cells

## Abstract

Phytohormones, specifically from the cytokinin family, represent a promising class of sustainable, non-immunogenic alternatives to animal-derived growth factors in skin tissue engineering. This review explores the innovative integration of selected cytokinins—namely kinetin (K), benzyladenine (BA), and traumatic acid (TA)—into biocompatible biomaterials, focusing on their distinct molecular mechanisms and regenerative capacities in human cell models. At the cellular level, these compounds comprehensively modulate cell fate, proliferation, and survival. Benzyladenine and its derivatives regulate cell cycle transitions, enhance D-type cyclin expression, and selectively inhibit phosphodiesterase-4 (PDE4), thereby suppressing tumor necrosis factor-alpha synthesis to deliver crucial intrinsic anti-inflammatory benefits. Concurrently, kinetin functions as an advanced molecular shield and potent free radical scavenger, delaying the morphological features of fibroblast senescence and protecting nuclear DNA against oxidative damage by preventing 8-oxo-dG formation. Furthermore, the plant wound hormone traumatic acid exhibits a dual biological activity; it selectively serves as a significant antioxidant that accelerates extracellular matrix remodeling by driving protein and collagen biosynthesis in normal skin fibroblasts, while shifting to a pro-oxidant, cytotoxic mechanism in cancerous cells. By merging these specialized anti-inflammatory, anti-aging, and matrix-stimulating pathways into biocompatible scaffolds or hydrogels, cytokinin-functionalized biomaterials eliminate the safety and contamination risks associated with mammalian cell products. Ultimately, this approach establishes a highly cost-effective, scalable, and multi-functional platform capable of driving advanced tissue repair, enhancing wound healing dynamics, and ensuring significant biocompatibility.

## 1. Introduction

**Figure 1:**
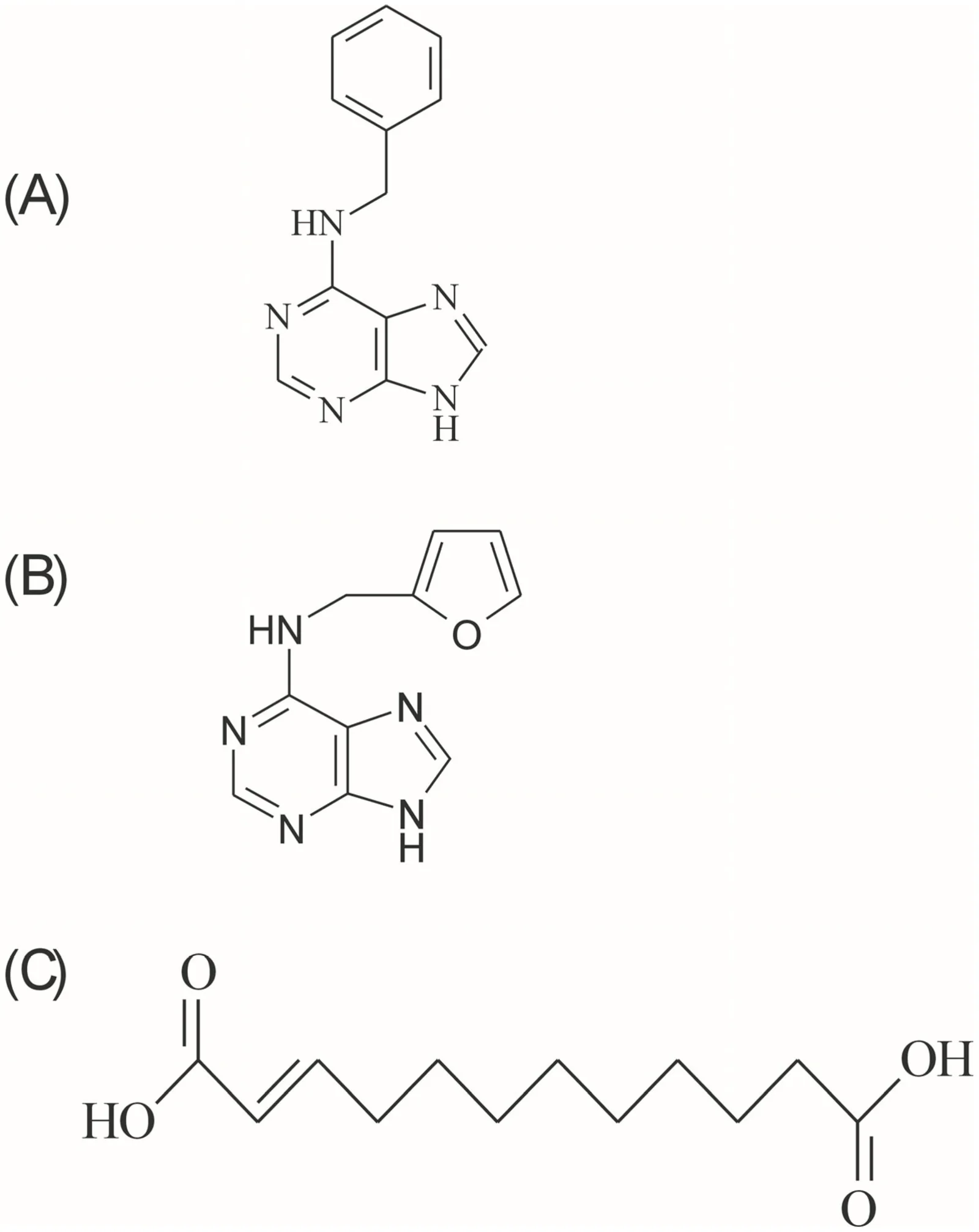
Chemical structure of (**A**) benzyladenine (BA), (**B**) kinetin (K), and (**C**) traumatic acid (TA).

Cytokinins are one of the main groups of plant hormones that stimulate anabolic processes, cell growth, and cell division. They stimulate cytokinesis, karyokinesis, replication, transcription, translation, and active transport across cell membranes. They have also been shown to inhibit the activity of enzymes that degrade nucleic acids and proteins, delaying cell aging [1]. Cytokinins also accelerate tissue regeneration and extend the cell development cycle. They stimulate the biosynthesis of nucleic acids and the formation of native protein structures. Cytokinins are derivatives of the purine base adenine, in which the amino group at carbon C-6 is most often attached to a five-carbon isoprene aliphatic chain, or less frequently, to an aromatic benzyl substituent in various chemical modifications [2,3,4]. In plants, the most common cytokinins are those with aliphatic substituents, such as zeatin, dihydrozeatin, and their various modifications with hydroxyl or methyl substituents. Cytokinins with aromatic benzyl substituents, such as benzyladenine (BA), are less common (Figure 1). This group of compounds also includes m- and o-topolin, i.e., m- and o-hydroxybenzyladenine. Synthetic analogues of adenine cytokinins are known, such as N6-farnesyladenine, N6-geranyladenine, and N6-furfuryladenine (kinetin-K), which is one of the most biologically active cytokinins. In plant cells, cytokinins usually occur as N-glycosidic linkages or as free bases [5,6].

Cytokinin properties are also exhibited by derivatives of unsaturated fatty acids, such as traumatic acid (TA, 1-deceno-1,10-dicarboxylic acid) and its aldehyde form—traumatin—as well as a compound such as allantoin, which is a product of partial degradation of purine bases (Figure 1) [7].

### 1.1. Characterization of Cytokinins as Modulators of Cell Fate

The first cytokinin discovered and, to date, the best-studied is kinetin (N-6-furfuryladenine) (Figure 1). It was first isolated from herring sperm DNA in 1955 and was long considered an artifact of DNA remodeling processes. In 1996, kinetin was found in commercially available DNA, in DNA freshly extracted from human cells, in plant cell extracts, and in human urine [8,9,10,11]. Experimental results indicate that kinetin is an important component of the metabolic pathway that allows cells to eliminate excess free radicals. Through this pathway, cells neutralize the harmful effects of free radical reaction products, such as furfural, and respond to oxidative stress by inducing defense and repair mechanisms [12,13]. The identification of kinetin, which until now had only been found as a synthetic compound in natural DNA molecules, raised questions about the mechanism of its formation at the nucleic acid level. The chemical structure of N-6-furfuryladenine suggests that the reaction of the adenine residue of DNA with furfural occurs only synthetically [14]. However, the method by which this aldehyde is synthesized has not yet been explained. Furfural is a dietary mutagen found in many foods and beverages (e.g., coffee, white bread) and is produced by the intense heating of sugars. It is also present in the gas phase of tobacco smoke in relatively large amounts (on the order of 45–110 mg per cigarette). Furfural has been identified in human blood plasma, urine, heart homogenate, and extracts from human osteoblast cultures [15,16].

**Figure 2:**
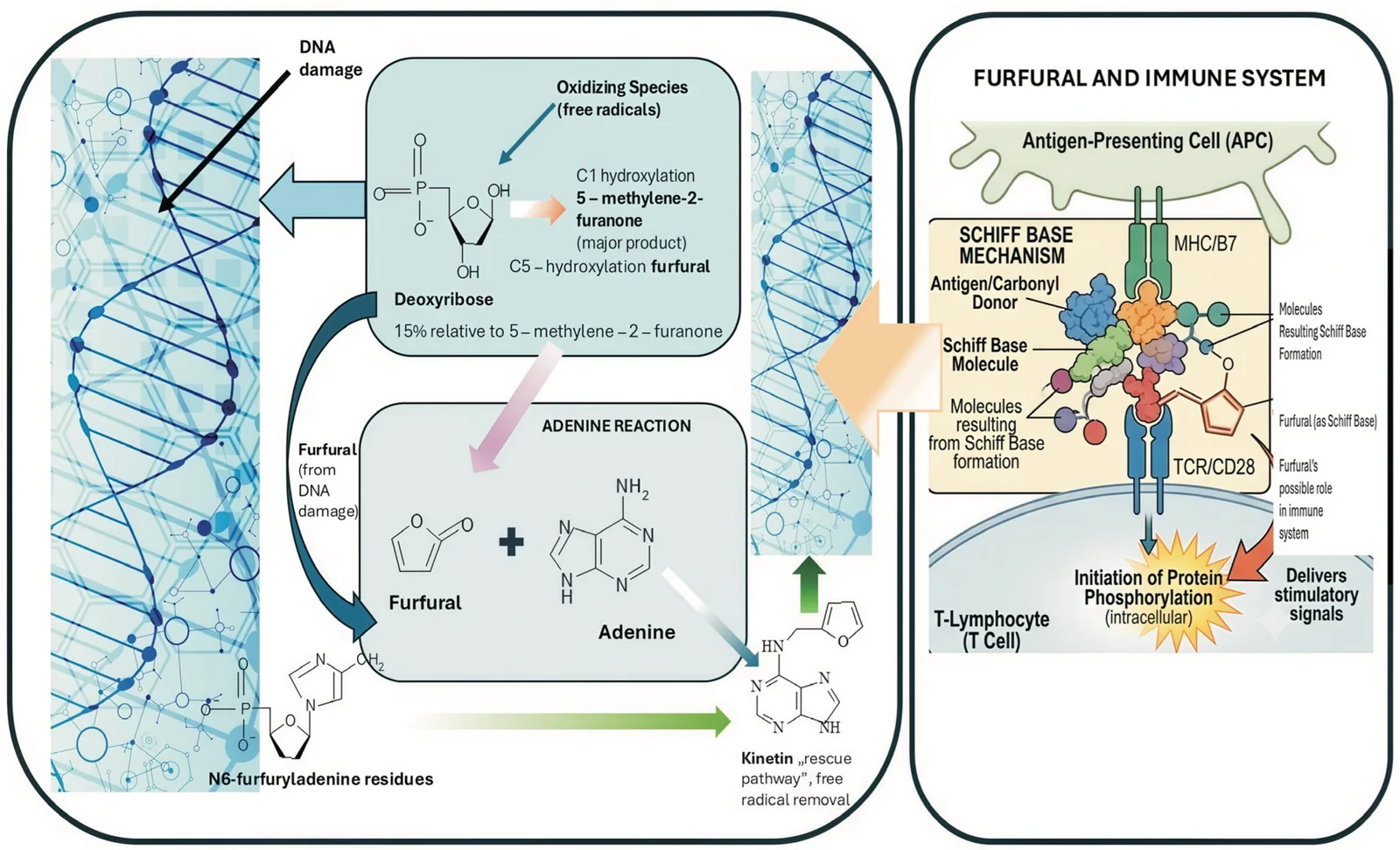
Deoxyribose oxidation, furfural formation and its role in immune stimulation.

Research results indicate that furfural, as an oxidized sugar moiety, is formed during DNA-damaging oxidation reactions (Figure 2). The primary target in the deoxyribose oxidation reaction is the anomeric carbon (C1), and its hydroxylation yields 5-methylene-2-furanone. Furfural has also been identified among the products of metalloporphyrin reactions with DNA [17,18]. The mechanism of this reaction suggests that furfural may be formed by hydroxylation at the C5 carbon of deoxyribose in DNA. The expected amount of furfural formed by C5 hydroxylation relative to 5-methylene-2-furanone (C1 hydroxylation) is 15%. These results indicate that furfural is one of the major products of free radical DNA damage reactions [14]. The formation of furfural, and subsequently kinetin from it, also serves as a kind of “rescue pathway” for the cell, enabling the removal of free radicals from the cell interior. The presence of furfural in combination with adenine residues as the first product of deoxyribose oxidation suggests that the degradation of sugar residues is one of the main pathways of DNA damage, in addition to the modification of nucleic acid bases. These observations are consistent with a new approach to the capabilities and therapeutic potential of the immune system, which considers the formation of Schiff bases. Molecules resulting from the formation of Schiff bases can act as carbonyl donors and can be expressed on the surface of antigen-presenting cells. Therefore, they interact with amines on receptor T cells and deliver stimulatory signals to T lymphocytes, which are necessary for the initiation of protein phosphorylation. Research is currently underway to elucidate the possible role of furfural, which is also a Schiff base, in the immune system [19,20]. However, it can be hypothesized that endogenously produced furfural may serve as a previously overlooked carbonyl signal of oxidative DNA damage. Furfural may influence the immune system’s recognition of oxidatively damaged cells. Furthermore, its conversion into kinetin could represent a protective mechanism that limits carbonyl stress and mitigates inflammation. Naturally, this requires further research, although the hypothesis appears quite plausible.

It has been suggested that furfural forms bonds with adenine in DNA, which is then dehydrated at low pH to form kinetin. This is consistent with previous observations of furfural reactions with DNA and AT-rich oligonucleotides, which destabilized DNA secondary structure by modifying DNA bases. The presence of N6-furfuryladenine in DNA indicates a direct link between deoxyribose oxidation and a new type of purine base modification. Kinetin is a byproduct of the oxidation reaction that results from the reaction of an adenine residue with furfural, which is a major product of free radical oxidation of DNA [21,22].

The primary impact of benzyladenine (BA) and its derivatives on cellular biochemistry is the stimulation of cell division. They actively control the G1/S and G2/M cell cycle checkpoints by enhancing the expression of CYCD3, the gene encoding D-type cyclins. By comparison, D-cyclin activity in animal cells is governed by multiple growth factors, which act as key drivers of the cell cycle, particularly during G1 phase regulation. [23].

A key property of BA-derived cytokinins is their ability to nonspecifically inhibit certain human protein kinases, including CDKs. CDKs regulate various phases of the eukaryotic cell cycle. Cell cycle regulation and tumor growth are closely linked. Abnormal expression or mutations in genes encoding cyclin-dependent kinases (CDKs), cyclins, or cyclin-dependent kinase inhibitors are found in many types of cancer [24,25]. An additional substituent at the C2 or N9 site of the BA molecule leads to potent and specific inhibition of various important protein kinases, such as CDK1/cyclin B, CDK/cyclin A, CDK2/cyclin E, brain CDK5/p35, and ERK1/MAP kinase. These factors have a high capacity to arrest certain phases of the cell cycle and induce apoptosis. CDK1 kinases play a role in mitosis, while the remaining CDKs play a role in the early phases of cell division (interphase). Control of these processes is especially important in cancer cells, where cell cycle regulators are often subject to various mutations. Disturbances in the apoptotic process lead to various diseases, including cancer. Therefore, the activity of CDKs and their potential inhibition is a new target for the development of new anticancer drugs [23,26].

Traumatic acid belongs to the oxygen derivatives of unsaturated fatty acids. It was first isolated from immature bean pods (Phaseolus vulgaris) in 1937–1938 by Bonnier and English. So far, its presence has been detected in mesophyll and meristem tissues of many plant species. Traumatic acid, as well as its aldehyde derivative—traumatin (2-dodeceno-1-al-10-carboxylic acid) are called wound hormones because they occur in large quantities around the injured area in plants and they stimulate cell division in the healing tissue. In the largest amounts both compounds are present in the young, intensively growing organs such as leaves, fruits and seeds. Traumatin, an aldehyde form, turned out to be a more biologically active compound than traumatic acid in plants. TA as a compound of vegetable origin could constitute an alternative to the fatty acids of animal origin [27].

Most likely, the main function of TA is to stimulate cell division [28,29]. Stimulation of cultures of various unicellular algae species with this compound results in a more than two-fold increase in cell number compared to the control. However, the molecular mechanism of TA action is not yet fully understood, and there is a lack of literature data on its effect on basic metabolic and physiological processes occurring in plant and animal cells. The only existing data indicate that TA enhances tyrosine phosphorylation in proteins by 1.5-fold [28]. The increase in the content of phosphorylated tyrosine residues is greatest for proteins with the following molecular masses: 19, 20, 22, 26, 31, 42, and 74 kDa. Macropeptides with molecular masses of 36, 47, and 49 kDa showed inhibited tyrosine phosphorylation after TA stimulation. This hormone can likely affect the activity of protein kinases and phosphatases, which is why intensification or inhibition of tyrosine phosphorylation in specific proteins is observed. Probably, as a result of TA binding to a specific receptor in the cell membrane, appropriate kinases are activated, which results in phosphorylation and conformational changes in the relevant proteins and initiation of further signal transduction cascade in the cell [28].

The process of ω-oxidation of fatty acids in animal cells is similar to the formation of traumatic acid (TA) in plants. The stimulating effect of TA at the molecular level in plant and animal cells is likely similar to that of non-esterified unsaturated fatty acids (NEFA). It is likely that traumatic acid, which is an oxygen derivative of unsaturated fatty acids (NEFA), may act similarly to the way unsaturated, non-esterified (free), and protein-bound fatty acids act at the molecular level in cells. Based on the literature, traumatic acid (TA)—a dicarboxylic acid and plant wound hormone historically known for causing cicatrization (healing) in plants—exhibits a fascinating dual or selective biological activity when introduced to human cells [7]. Depending on whether the target cells are healthy or cancerous, TA triggers completely contrasting metabolic and oxidative pathways. In normal human cells, particularly human skin fibroblasts, traumatic acid acts as an important antioxidant and stimulating agent [7]. Conversely, when tested on human cancer models (such as MCF-7 and ZR-75-1 human breast cancer cell lines), traumatic acid flips its mechanism of action to act as a pro-oxidant and cytotoxic agent [30,31].

### 1.2. Biologically Active Biomaterials: The Concept of Using Plant Hormones in Skin Tissue Engineering as Safe Alternatives to Animal Growth Factors

The use of plant hormones (phytohormones) in skin tissue engineering as functional components of biomaterials would allow for the replacement of animal-derived growth factors with sustainable, non-immunogenic alternatives. This simultaneously avoids side effects and eliminates the problems posed by animal-derived components or synthetic compounds. Unlike animal-derived growth factors, plant hormones eliminate the risk of zoonotic disease transmission, immunogenicity, and toxic contamination (e.g., bacterial endotoxins). The possibility of using plant-derived substitutes has already emerged in stem cell culture for therapeutic purposes, where growth factors and serum are essential for maintaining stem cell characteristics in culture and for inducing differentiation. However, it should be noted that growth factors are primarily produced in bacterial (*Escherichia coli*) or animal cells, and their use raises safety concerns that must be addressed before stem cell therapy can be commercialized. To address this issue, research has been conducted on plant-produced proteins [32]. Phytohormones such as auxins, cytokinins, and gibberellins can be incorporated into biocompatible hydrogels or nanofibrous scaffolds to stimulate cell proliferation, promote angiogenesis, and accelerate tissue repair. Another advantage of plant-derived components is that plant biomaterials and plant-derived recombinant proteins are significantly more cost-effective and can be rapidly scaled up compared to mammalian cell culture. Furthermore, phytohormones often provide intrinsic antioxidant and anti-inflammatory properties, increasing the overall biocompatibility of the scaffold [33,34].

### 1.3. The Aim of the Study

The aim of this study is to analyze the potential use of selected plant hormones from the cytokinin group as key components of biologically active biomaterials. The concept of using plant hormones in skin tissue engineering as safe alternatives to animal growth factors is a novel idea stemming primarily from the highly beneficial properties and high safety level of these compounds, which they demonstrate in in vitro studies on human cells. The aim of this study is to comparatively analyze the effects of TA, K, and BA on the structural integrity of human skin cells and their anti-aging abilities when exposed to modern medical materials.

## 2. Molecular Mechanisms of Biological Activity

### 2.1. Stimulation of Cell Division and Cell Growth by Plant Hormones from the Cytokinin Group

#### 2.1.1. The Influence of Cytokinins on the Cell Cycle and the Biosynthesis of Nucleic Acids and Proteins

Cytokinins in cells influence karyokinesis, primarily in the G2 stage of interphase, and stimulate nucleic acid biosynthesis. They stimulate the activity of polymerases and transferases involved in nucleic acid biosynthesis during replication and transcription. Due to their chemical structure, some cytokinins can be incorporated near the tRNA anticodon or in its side loop, as well as in the polyadenylate fragment of mRNA. They accelerate the correct reading of codons found in mRNA by complementary anticodons located in aminoacyl-tRNA, which is crucial for their stimulatory activity [35,36,37]. Cytokinins also activate enzymes such as aminoacyl synthetase and aminoacyltransferase, thereby stimulating the translation process, especially during the initiation and elongation stages. They also contribute to the formation of the native structure of proteins, which determines their biological activity. They increase the number of ribosomes and polysomes, their translational activity, and the activity of the endoplasmic reticulum. As a result, the number of proteins increases, especially enzymatic, regulatory, and transport proteins, and those playing an important role in cell metabolism. Simultaneously, the activity of enzymes that degrade proteins (proteases), nucleic acids (ribonucleases and deoxyribonucleases), as well as enzymes such as oxidases and phosphatases, is reduced [38].

Benzyladenine (BA) and its derivatives regulate many biochemical processes at the cellular level, but their greatest impact is on cell division (Table 1). In the cell cycle, they are involved in regulating the G1/S and G2/M phase transitions. They increase the expression of the CYCD3 gene, which encodes D-type cyclins. In animal cells, D-cyclin activity is regulated by many growth factors, which play a key role in regulating the cell cycle, especially the G1 phase [39,40].

Considering the influence of cytokinins on the cell cycle, it is important to emphasize the ability of BA derivatives to nonspecifically inhibit certain human protein kinases, including CDKs (Cyclin-Dependent Kinases). CDKs are regulators of various phases of the eukaryotic cell cycle. An additional substituent at the C2 or N9 site of the BA molecule leads to the emergence of strong and specific inhibition of various important protein kinases, such as CDK1/cyclin B, CDK/cyclin A, CDK2/cyclin E, brain CDK5/p35, and ERK1/MAP kinase. These factors have a high capacity to arrest certain phases of the cell cycle and induce apoptosis. CDK1 kinases play a role in mitosis, while the remaining CDKs play a role in the early phases of cell division (interphase). Control of these processes is important in cells, as disruptions lead to various diseases [24,41].

Traumatic acid is an example of a cytokinin-like compound, which was found to be highly effective in stimulating protein biosynthesis, particularly collagen (Table 1). It increased total protein and collagen biosynthesis, increasing its secretion into the cellular matrix, suggesting potential applications in wound healing and the treatment of skin diseases, but also indicating its potential use as a component of biomaterials with additional beneficial functions [7].

Rather than acting solely as a direct up-regulator of synthesis, kinetin operates extensively as a molecular shield to preserve existing protein and nucleic acid pools (Table 1). Kinetin protects long-lived proteins (such as histones and extracellular matrix structural components) from glycoxidative cross-linking and damage induced by reducing sugars like ADP-ribose and it is highly effective at reducing oxidative and genotoxic damage to nuclear DNA caused by reactive oxygen species [12].

#### 2.1.2. The Role of Kinetin in Delaying the Morphological Features of Fibroblast Aging (Maintaining the Shape and Size of Cells Typical of Young Tissues)

**Figure 3:**
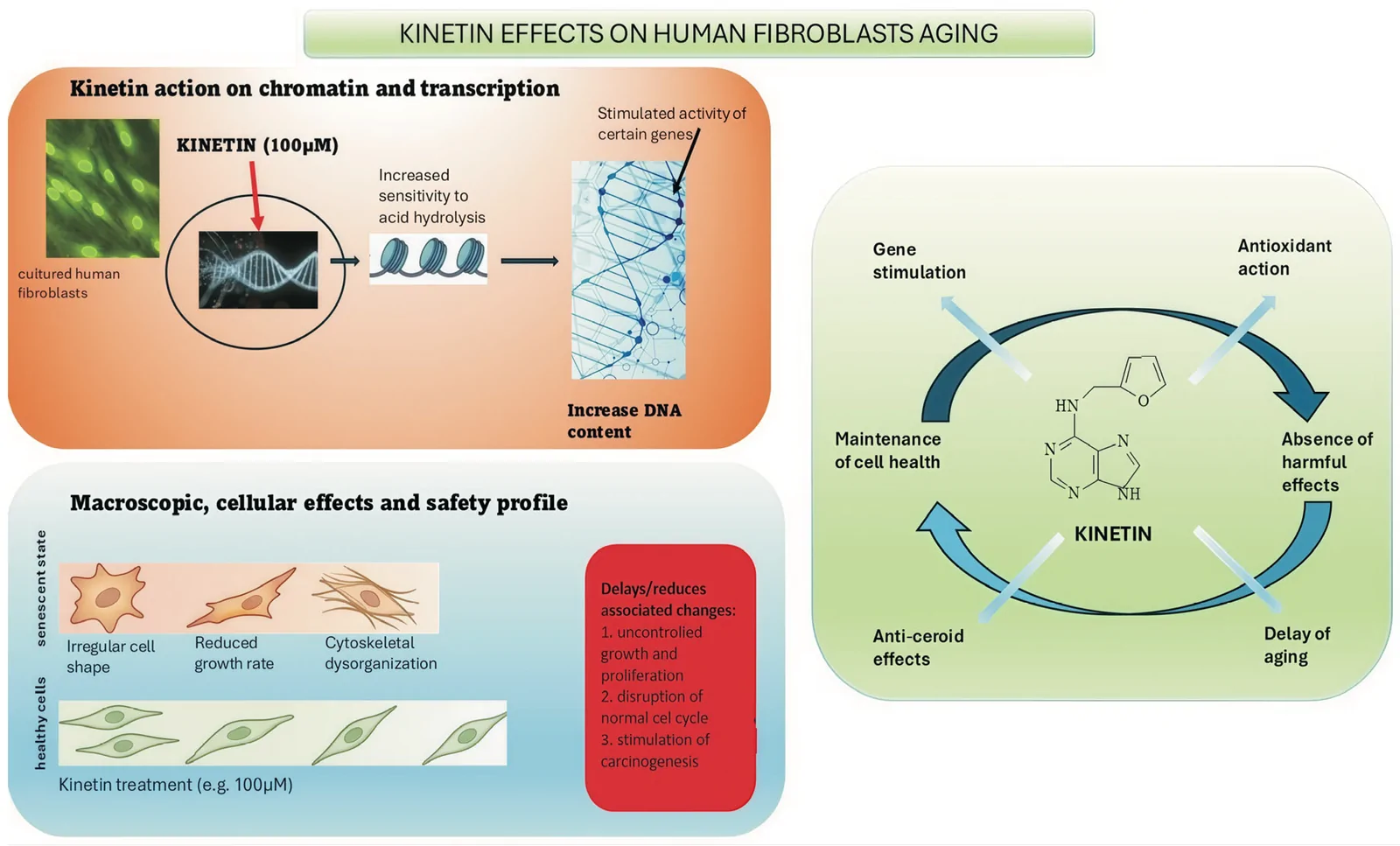
Kinetin influence on human fibroblast aging.

Kinetin stimulates the activity of certain genes in human fibroblast cultures (Table 1) (Figure 3). The changes observed after its stimulating effect primarily include increased sensitivity of chromatin to acid hydrolysis, which is associated with increased transcriptional activity and increased DNA content in the fibroblast cell nucleus. Kinetin at a concentration of 40–200 μM delays characteristic cellular and biochemical changes associated with aging in fibroblast cell cultures [8,39,42,43]. These changes are related to kinetin’s influence on cell shape, growth rate, cytoskeletal structure, macromolecular synthesis, and lipofuscin content. The accumulation of lipofuscin and ceroids in cells is associated with the age-related imbalance between ROS generation and the cell’s antioxidant capacity. The predominance of oxidative processes causes the accumulation of oxidized proteins and lipids, as well as glycation products such as pentosidine. The anticeroid effects of kinetin may stem from its antioxidant properties. All these changes occur without any potentially harmful effects, such as disruption of the normal cell life cycle, uncontrolled cell growth and proliferation, or stimulation of carcinogenesis [44,45].

The anti-aging effects of kinetin were also confirmed in dermal endothelial cells. Kinetin administration increased the expression of myosin and actin, cytoskeletal proteins. Cytokinin also activated cell proliferation, delayed endothelial cell aging, and stimulated their proliferative and metabolic capabilities [46].

### 2.2. Cell Signaling and Transduction

#### The Effect of BA on Specific Metabolic Pathways Regulating the Rate of Cell Proliferation

Cytokinins from the adenine derivative group, such as the N-9-substituted derivative, exhibit significant inhibition of phosphodiesterases (PDEs), particularly phosphodiesterase-4 (PDE4). PDEs are widely distributed in mammalian tissues, where they play a central role in cellular signal transduction through the hydrolysis of cAMP and cGMP. Their diversity allows for specific distribution at the cellular and subcellular levels. Therefore, PDEs can selectively regulate various biochemical cellular functions. The important role played by PDEs in intracellular signal transduction opens the possibility of their use as therapeutic agents in inflammatory processes. The PDE superfamily consists of 11 families (PDE1 to PDE11) [47,48,49].

PDE4 (cAMP–PDE), previously called ROI-PDE or PDE-IV, is primarily found in the brain, inflammatory cells, cardiac muscle, blood vessels, and smooth muscle. It is the largest family of PDEs. It is encoded by four different genes (PDE4A, PDE4B, PDE4C, PDE4D), and alternative mRNA splicing produces various PDE4 isozymes (at least 35 different proteins). The PDE4 family hydrolyzes only cAMP and contains a unique region with an amino acid sequence called UCR1 and UCR2 (Upstream Conserved Region 1 and 2). These regions are located between the amino terminal end and the catalytic region of PDE4 enzymes [50]. PDE4 activity is regulated by phosphorylation, protein association, endogenous factors, and proteolysis. Prolonged cAMP accumulation induces long-term regulation of PDE4, particularly its activation [51]. Benzyladenine derivatives have been shown to be selective PDE4 inhibitors. PDE4 inhibition is associated with the anti-inflammatory properties of BA derivatives. They also significantly inhibit the synthesis of TNF-α (Tumor Necrosis Factor). This factor is a key cytokine contributing to the development of inflammatory diseases. High levels of TNF-α are observed in various pathological conditions, such as asthma, rheumatoid arthritis, and inflammatory bowel disease. PDE4 inhibitors are anti-TNF-α agents that inhibit its synthesis and are used in many inflammatory diseases, such as asthma, chronic obstructive pulmonary disease, atopic dermatitis, rheumatoid arthritis, and various neurological diseases [52,53]. Therefore, it seems that the use of BA derivatives as components of biomaterials could provide an additional anti-inflammatory effect, which would further support wound healing and reduce the risk of inflammation at the site of biomaterial application.

## 3. Advanced Antioxidant Protection and Stability of Biomaterials

### 3.1. Kinetin as a Free Radical Scavenger

#### 3.1.1. The Unique Ability of Kinetin to Inhibit the Formation of 8-oxo-dG (A Marker of DNA Damage

Kinetin exhibits antioxidant properties both in vitro and in vivo, which can also be used as an additional advantage in the construction of biomaterials with additional properties. It protects cell DNA against oxidative damage caused by the accumulation of 8-oxo-dG produced as a result of the Fenton reaction. It inhibits the formation of 8-oxo-dG in a dose-dependent manner, with a maximum effect at a concentration of 100 μM (Table 1) [54].

The most commonly observed modification in DNA, considered a marker of oxidative damage to genetic material, is 8-oxo-dG. This modification is produced, among other things, by the Fenton reaction. This type of reaction primarily generates hydroxyl radicals (•OH) [55]. Kinetin has been found to significantly inhibit the formation of 8-oxo-dG. This process may occur via two metabolic pathways. First, kinetin may prevent the formation of free hydroxyl radicals, and second, it may prevent these free hydroxyl radicals from reacting with DNA. It may bind iron, which then cannot be used as a reducing agent in the Fenton reaction and cannot be bound by DNA. Kinetin may behave as a free radical scavenger in two different ways, as free radicals can either directly bind hydrogen from the kinetin amine bond carbon or undergo rapid dismutation, which results from the SOD-like activity of the metal-kinetin complex. Kinetin forms complexes with copper(II), which have SOD-like activity. In biological systems, O_2_- ions play a dual role in ROS generation: through the Fenton reaction, where they are converted to H_2_O_2_, but also by acting as reducing agents for free iron ions or iron-binding proteins that deliver iron to the Fenton reaction. Thanks to the activity of kinetin and Cu(II) complexes, excess O_2_- ions are removed, leaving less Fe2+ available for the Fenton reaction [54,56].

#### 3.1.2. Prevention of Oxidation of Membrane Lipids, Which Is Crucial for the Durability of the Biomaterial at the Site of Implantation/Application

Kinetin and benzyladenine exhibit significant protective effects against lipid peroxidation in mammalian and human cells. Kinetin primarily acts as a strong antioxidant agent that shields cellular membranes from oxidative degradation. Kinetin significantly decreases membrane phospholipid peroxidation and exhibits protective properties by lowering MDA production [39]. It stimulates key antioxidant enzymes like superoxide dismutase (SOD) and catalase, which neutralize the free radicals responsible for initiating lipid damage [1,39]. Kinetin prevents the depletion of internal cellular thiol groups and helps maintain high levels of reduced glutathione (GSH), reinforcing the cell’s capacity to halt lipid chain reactions [12].

Traumatic acid (TA) exhibits a highly selective, dual influence on lipid peroxidation depending entirely on the nature of the target cell. It acts as an antioxidant shield in healthy human cells, but shifts into a pro-oxidant weapon when introduced to malignant human cancer cells. In normal human cell lines, particularly human skin fibroblasts, traumatic acid significantly decreases lipid peroxidation [39]. Lipid peroxidation occurs when reactive oxygen species (ROS) attack the polyunsaturated fatty acids in cell membranes, creating toxic degradation products like malondialdehyde. TA successfully reduces the generation of ROS and subsequently lowers MDA levels. By suppressing the oxidative chain reactions that degrade lipid bilayers, TA protects cell membrane integrity and supports healthy cellular proliferation and collagen synthesis.

### 3.2. Synergy in Enzymatic and Nonenzymatic Defense

#### 3.2.1. Combined Action of TA and K in the Activation of Enzymes and Oxidative Stress Parameters

When evaluated in human cellular models, both kinetin (an adenine-type cytokinin) and traumatic acid (a dicarboxylic acid wound hormone) function as important modulators of cellular redox status. While they belong to different chemical classes, both compounds share the significant ability to reinforce the endogenous antioxidant defense systems of healthy human cells, while exhibiting concentration-dependent or cell-specific pro-oxidant profiles.

Kinetin may affect cellular metabolism in two ways. First, it may induce the synthesis of repair enzymes, which remove modified bases from the DNA chain and are involved in protection against free radicals and oxidative stress. Secondly, it forms complexes with copper (II) ions, which activate superoxide dismutase. Kinetin stimulates SOD activity, enhancing the dismutation of superoxide radicals into hydrogen peroxide and upregulates CAT activity, accelerating the breakdown of H_2_O_2_ into water and molecular oxygen [39,57].

Traumatic acid’s influence on oxidative parameters is uniquely dependent on the metabolic state of the cell (selective cytotoxicity). In normal fibroblasts TA acts as a protective agent. By activating the TGF-beta/Smad signaling pathway, it not only stimulates collagen synthesis but simultaneously lowers baseline oxidative stress, establishing a highly favorable microenvironment for cell viability and wound healing [7].

For both compounds, the cytoprotective, antioxidant effect is highly concentration-dependent. While low doses stimulate healthy cellular defenses, supra-physiological concentrations (500 μM) of both kinetin and traumatic acid can overwhelm cellular homeostatic pathways, leading to a pro-oxidant state and cytostatic effects.

#### 3.2.2. Glycation Inhibition: The Role of Kinetin in Preventing the Formation of Glycation End Products (AGEs), Which Protect Collagen and Elastin in the Skin Structure

Kinetin also inhibits the formation of pentosidine, one of the products of non-enzymatic protein glycation, and significantly reduces protein aggregation during glycation. Therefore, it is considered a potent inhibitor of protein and nucleic acid oxidation and glycosylation, especially in vitro [58]. Oxidized proteins are an important factor in the aging process because they are products of glycation and oxidation, which significantly accumulate in cells and tissues during aging. Glycation leads to the binding of sugar residues to free amino acid or nucleotide groups, while glycoxidation involves successive glycation and oxidation reactions [59,60]. Glycoxidation processes occurring outside the cell are slow because their rate depends on the concentration of glucose, the least reactive sugar. Intracellular processes, however, occur much faster because they are dependent on the increase in the intracellular concentration of more reactive glycation factors than glucose, such as pentoses (e.g., ribose and arabinose) and alpha-oxoaldehydes (e.g., glyoxal). These processes result in the formation of advanced glycation/glycoxidation end products (AGEs), such as pentosidine [61,62]. Kinetin has been found to inhibit slow and fast glycation and glycoxidation processes. It also inhibits protein oxidation and aggregation, acts as an antioxidant, catalyzes SOD-mediated reactions, and protects against ascorbate-induced oxidative stress. Both kinetin and adenine exhibit similar antioxidant activity, but kinetin is more effective. The furfuryl substituent at the N6 position appears to be important for maintaining kinetin’s protective properties. Paradoxically, this substituent is responsible for ROS-induced damage and, at the same time, appears to be responsible for kinetin’s antioxidant properties [58,63].

## 4. Designing Intelligent Biomaterial Structures

The construction of biomaterials for medical scaffolds is one of the pillars of tissue engineering and regenerative medicine. Their primary purpose is to temporarily mimic the natural extracellular matrix (ECM), allowing cells to adhere, proliferate, differentiate, and ultimately reconstruct damaged tissue [64,65].

Creating an effective scaffold requires precise completion of several key technological steps. The first is the selection of the material base. Natural polymers (e.g., collagen, chitosan, alginate, hyaluronic acid) are chosen for their excellent bioactivity, synthetic polymers (e.g., PLA, PGA, PCL, PLGA) for mechanical stability, or inorganic materials (e.g., hydroxyapatite, bioglasses) for bone tissue regeneration. The next step is designing the spatial architecture. The scaffold must have high porosity (usually above 80–90%) with an interconnected pore structure. This allows for the free diffusion of oxygen and nutrients, cell migration, and neovascularization (the formation of new blood vessels). The material is then processed using advanced engineering techniques to obtain the desired 3D geometry, and the finished scaffold is cross-linked (e.g., physically using UV or chemically, e.g., EDC/NHS) to optimize biodegradation time, and sterilized using methods that do not degrade the polymer structure (e.g., low-temperature plasma or ethylene oxide) [66,67].

Classic scaffolds function only as passive scaffolds. Contemporary biomaterials engineering focuses on active (intelligent) scaffolds that also function as local drug delivery systems (scaffold-based drug delivery systems). The introduction of anti-inflammatory substances aims to mute the foreign body response (FBR) and control macrophages at the implantation site (promoting macrophage polarization from the pro-inflammatory M1 phenotype to the regenerative M2) [68,69].

### 4.1. Cytokinins in Tissue Scaffolds

Taking into account the above and the analysis of the properties of the described cytokinins, including the biocompatibility of K and BA—lack of toxicity and protective effect against mutagenic factors—it can be concluded that these compounds have a real potential for use as bioactive ingredients in biomaterials for medical purposes.

BA appears to be a good candidate for surface modification of materials to enhance cell adhesion. Furthermore, in fibroblast cultures, benzyladenine derivatives have been observed to induce cell elongation, attenuate their movement, and dramatically increase their adhesion to the substrate. A similar effect on cell shape, movement intensity, and adhesion was observed when cultures were stimulated with PDE inhibitors, which increased intracellular cAMP levels [70].

To harness the therapeutic and biological potential of kinetin, benzyladenine (BA), and traumatic acid in tissue engineering and regenerative medicine, their efficient incorporation into functional biomaterial matrices is crucial. The incorporation of these bioactive molecules into polymer scaffolds, hydrogels, or electrospun meshes can be achieved using several different biofabrication techniques [71,72]. The first of these is physical entrapment. Due to the small size of their molecules, kinetin, BA, and traumatic acid can be directly dissolved in prepolymer solutions prior to the gelation process (e.g., ionic cross-linking of alginate or thermal gelation of gelatin or collagen). This technique preserves the natural biological activity of these molecules without the need for harsh chemical reagents. Another technique involves electrospinning and core–shell structures. In the case of fibrous tissue scaffolds (e.g., made of polycaprolactone or a copolymer of lactic and glycolic acids), co-dissolution or coaxial electrospinning enables the encapsulation of compounds within the fiber core, protecting them from immediate leaching [73]. Another technique involves covalent immobilization and surface adsorption. Traumatic acid possesses two carboxyl groups, making it a good candidate for conjugation—using EDC/NHS chemistry—with matrices rich in amino groups, such as chitosan or gelatin. Meanwhile, the ring structures of kinetin and BA (purine derivatives) enable surface functionalization via hydrophobic interactions or hydrogen bonding on porous ceramic and polymeric surfaces.

Unlike labile protein growth factors (e.g., VEGF, FGF-2)—which easily denature under physiological conditions or during manufacturing processes—purine-derived cytokinins (kinetin and BA) exhibit significant chemical and thermal stability [74]. They are resistant to mild organic solvents and the ambient temperatures typical of scaffold fabrication processes. In contrast, traumatic acid—a derivative of a monounsaturated dicarboxylic fatty acid—is susceptible to photochemical and oxidative degradation. Therefore, to preserve the structural integrity of formulations containing traumatic acid, it is advantageous to conduct processes under an inert gas atmosphere or to employ co-encapsulation with biocompatible antioxidants (e.g., ascorbic acid, alpha-tocopherol).

A major challenge associated with small molecules is their tendency to undergo rapid initial release via passive diffusion. Strategies to achieve the sustained release required for tissue regeneration are therefore crucial [75]. Kinetin, BA, or traumatic acid can first be encapsulated in micro- or nanoparticles (e.g., PLGA microspheres or solid lipid nanoparticles) and subsequently dispersed within a hydrogel network. Alternatively, matrix-degradation-dependent release can be employed. Covalently bound or affinity-bound compounds rely on scaffold erosion—induced either enzymatically or through hydrolysis—enabling sustained release that is directly linked to host tissue remodeling.

**Figure 4:**
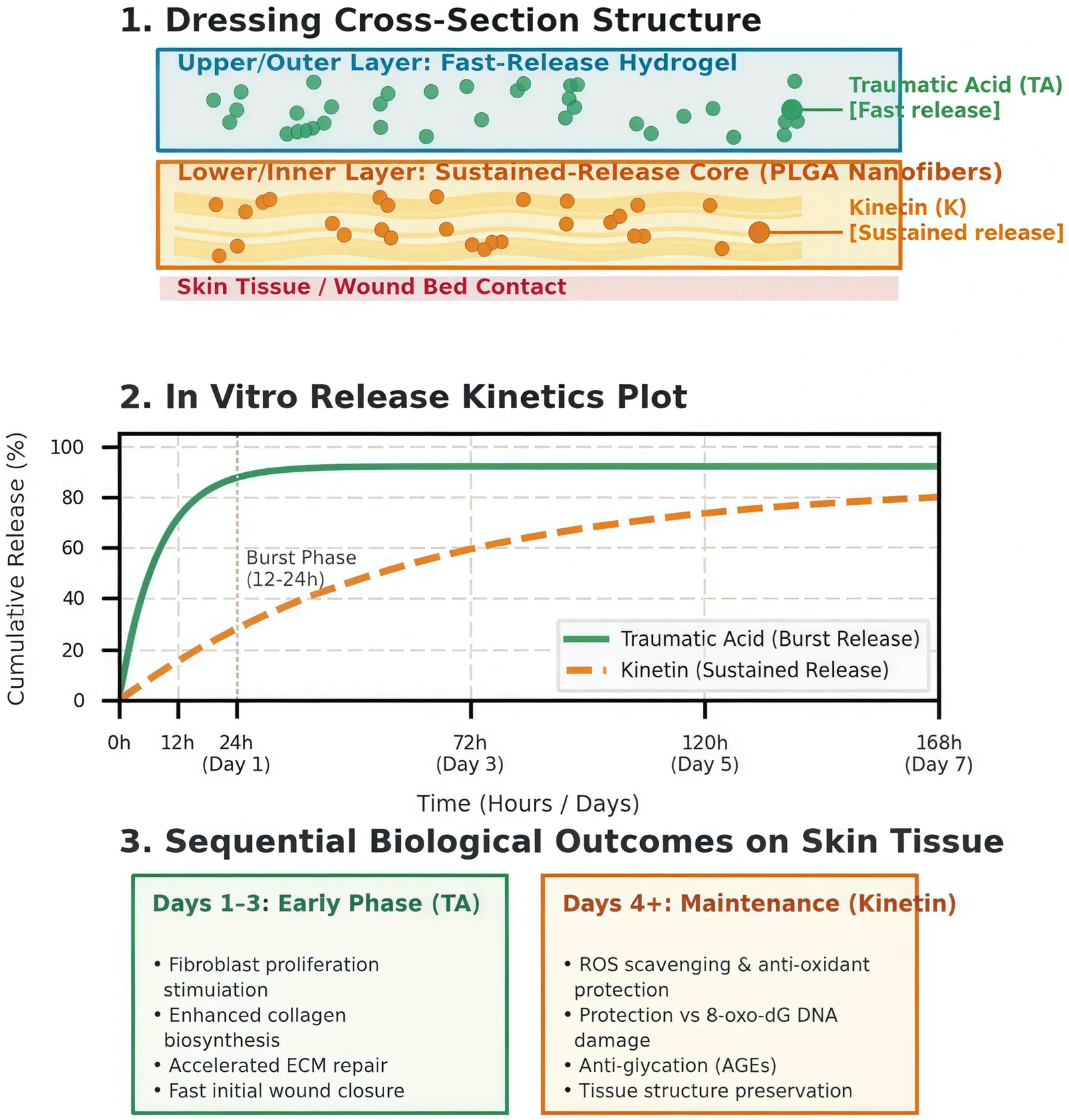
Conceptual framework of a multi-layered smart dressing for sequential phytohormone delivery in wound healing.

To harness these biological mechanisms in clinical practice, it is worth considering the development of a biphasic “smart dressing” designed for the sequential release of phytohormones (Figure 4). During the initial inflammatory and proliferative phases of wound healing, the rapid release of traumatic acid (TA)—from a hydrophilic outer matrix or a rapidly dissolving hydrogel layer—activates local protein and collagen synthesis, thereby accelerating early regeneration and wound closure. Subsequently, as the wound transitions into the maturation phase, the slower, sustained release of kinetin (K)—encapsulated in controlled-degradation polymer micro- or nanoparticles or within nanofibers—ensures lasting cytoprotection. Kinetin continuously limits oxidative damage, prevents the formation of 8-oxo-dG in DNA, and inhibits protein glycoxidation (the formation of AGEs), thereby protecting newly synthesized collagen fibers and preserving the functional viability of regenerating fibroblasts.

### 4.2. Applications in Regenerative Medicine and Cosmetology

A question worth considering is the potential use of K and BA in dermatological preparations and dressings that accelerate wound healing (e.g., in the treatment of rosacea or sun damage). Kinetin is helpful in repairing skin damage caused by excessive exposure to UV radiation. It improves skin structure, causes collagen and elastin fibers to regain their proper density, and reduces excessive skin pigmentation. Even very low doses of kinetin can be dermatologically effective, and its effectiveness depends largely on the duration of use. Studies confirm that this hormone improves the barrier function of the stratum corneum, reduces transepidermal water loss (TEWL), and improves skin tone, also by reducing existing discoloration. This compound has been shown not to cause allergies or skin lesions, even in sensitive skin [76,77,78,79].

### 4.3. Cytokinin Application Limitations

Although the incorporation of kinetin (K), benzyladenine (BA), and traumatic acid (TA) into biomaterial matrices offers a promising and cost-effective alternative to animal-derived growth factors, it is essential to consider certain key safety parameters and methodological limitations.

The biological activity and efficacy of these compounds are largely concentration-dependent. While low physiological doses of kinetin and traumatic acid promote cytoprotection, cell proliferation, and collagen synthesis, high, supraphysiological concentrations (e.g., 500 µM) shift the system toward a pro-oxidative state, resulting in cytostatic effects and cytotoxicity in normal human cells. Therefore, maintaining local concentrations within a narrow therapeutic window—using controlled-release systems—is crucial for preventing adverse cellular reactions.

Traumatic acid exhibits a dual biological mechanism: in healthy skin fibroblasts, it acts as an antioxidant, whereas in cancer models (e.g., MCF-7 and ZR-75-1 breast cancer cell lines), its activity shifts to pro-oxidant and cytotoxic. While this dual activity is advantageous for anticancer applications, it also highlights the need for detailed biocompatibility studies using various types of primary cells to rule out the risk of unintended tissue damage or reduced cell viability within the complex wound environment.

Purine cytokinins (kinetin and BA) exhibit high thermal and chemical stability during the scaffold fabrication process, whereas unsaturated fatty acid derivatives, such as traumatic acid, are highly susceptible to photochemical and oxidative degradation. This sensitivity limits the choice of processing techniques and necessitates the use of protective measures—such as encapsulation in an inert gas atmosphere or combination with antioxidants (e.g., ascorbic acid)—to prevent the loss of biological activity during storage and implantation.

Benzyladenine derivatives non-specifically inhibit human protein kinases, including cyclin-dependent kinases (CDKs). Although this property offers anti-inflammatory benefits through PDE4 inhibition and TNF-α suppression, uncontrolled CDK inhibition can disrupt the normal cell cycle (G1/S and G2/M transitions) or induce unwanted apoptosis in healthy, regenerating tissues.

It should also be mentioned that current evidence confirming the biological activity, anti-aging effects, and matrix remodeling capabilities of kinetin, BA, and TA stems primarily from in vitro cell models. Further in vivo preclinical studies are required to evaluate scaffold degradation, the foreign body response (FBR), the immune response, and long-term release kinetics within a complex tissue environment.

## 5. Conclusions

It appears that combining traumatic acid with kinetin and benzyladenine in biomaterials may offer a comprehensive approach to skin regeneration: from stimulating cell division (TA, BA) to protecting genetic material and protein structures (K). In vitro studies confirm that purine cytokines are potent inhibitors of cellular aging processes, making them appropriate components of anti-aging biomaterials. Incorporating these compounds into materials engineering would allow for the creation of structures with high bioactivity and a safety profile better than that of synthetic growth stimulators. Future research should focus on creating a “smart dressing” that sequentially releases TA (growth stimulation) and K (antioxidant protection). This combination of plant hormones could promote safe and rapid damage repair.

## Figures and Tables

**Table 1:** Summary of the main findings for K (kinetin), BA (benzyladenine) and TA (traumatic acid).

Compound	K	BA	TA
Cell model/target	Human skin fibroblasts	Human eukaryotic/somatic cell models	Human skin fibroblasts
	Human dermal endothelial cells	Human skin fibroblasts	Unicellular green algae (*Chlorella vulgaris*)
	SV40-transformed human fibroblasts	Human cancer cell models	Human breast cancer cell lines (MCF-7, ZR-75-1)
Effective concentration range	40–200 µM (delays cellular aging)	Described as dose-dependent; high doses induce cell cycle arrest	Low/physiological doses (stimulate proliferation/synthesis)
	Up to 100 µM (maximum 8-oxo-dG inhibition)		
	Cytotoxic at 500 µM	0.1–1 µM acts less on halting division in the fibroblasts	500 µM (supra-physiological dose causing pro-oxidant/cytostatic effects)
Key biological effects	Delays morphological and biochemical features of cell aging (maintains cell size, shape, and cytoskeletal integrity)	Regulates cell cycle progression and cell division	Stimulates cell division (>2-fold increase in cell counts) and tissue repair/wound healing
	Reduces accumulation of lipofuscin, ceroids, and glycoxidation products (pentosidine)	Inhibits synthesis of pro-inflammatory TNF *α*	Increases total protein and collagen biosynthesis/secretion into the extracellular matrix
	Promotes cell proliferation and metabolic capacity	Induces cell elongation, reduces motility, and increases cell adhesion to substrates	Serves as an antioxidant shield, lowering ROS production and membrane lipid peroxidation (MDA levels)
	Protects nuclear DNA and long-lived proteins from oxidative damage, glycoxidation, and cross-linking	Exhibits anti-aging, antioxidant, and antiproliferative/apoptotic activities in cancer cells	Shifts to a pro-oxidant, cytotoxic agent inducing cell death.
Proposed molecular mechanisms	Functions as a free radical scavenger and forms Cu(II)–kinetin complexes with SOD-like activity to convert superoxide radicals ($O_{2}^{∙-}$) and break down $H_{2}O_{2}$ via CAT	Regulates G1/S and G2/M cell cycle phase transitions by increasing *CYCD3* gene expression (D-type cyclins)	Binds cell membrane receptors to modulate protein kinase and phosphatase activity, regulating tyrosine phosphorylation
	Prevents Fenton reaction-driven 8-oxo-dG DNA damage by scavengin ∙OH radicals or chelating iron $(Fe^{2+}$)	Nonspecifically inhibits human protein kinases/CDKs (CDK1/cyclin B, CDK2/cyclin E, CDK5/p35, ERK1/MAPK)	Activates the TGF-*β*/Smad signaling pathway to drive collagen synthesis while suppressing baseline oxidative stress

## Data Availability

The data presented in this study are available on reasonable request from the corresponding author.
